# The Potential Application of Natural Clinoptilolite-Rich Zeolite as Support for Bacterial Community Formation for Wastewater Treatment

**DOI:** 10.3390/ma15103685

**Published:** 2022-05-20

**Authors:** Lacrimioara Senila, Alexandra Hoaghia, Ana Moldovan, Iulia Anamaria Török, Dalma Kovacs, Dorina Simedru, Calin Horea Tomoiag, Marin Senila

**Affiliations:** 1 Research Institute for Analytical Instrumentation Subsidiary, National Institute for Research and Development of Optoelectronics Bucharest INOE 2000, 67 Donath Street, 400293 Cluj-Napoca, Romania; lacri.senila@icia.ro (L.S.); alexandra.hoaghia@icia.ro (A.H.); ana.moldovan@icia.ro (A.M.); iulia.torok@icia.ro (I.A.T.); dalma.kovacs@icia.ro (D.K.); dorina.simedru@icia.ro (D.S.); 2 Faculty of Materials and Environmental Engineering, Technical University, 103-105 Muncii Boulevard, 400641 Cluj-Napoca, Romania; 3 Tomas Prodimpex SRL, 18 B-dul Eroilor Street, 400129 Cluj-Napoca, Romania; filtretomas@gmail.com

**Keywords:** natural zeolites, wastewater, support medium, microbial community, nitrification

## Abstract

The aim of this study was to investigate the use of natural zeolite as support for microbial community formation during wastewater treatment. Scanning electron microscopy (SEM), thermal decomposition and differential thermogravimetric curves (TGA/DGT) techniques were used for the physicochemical and structural characterization of zeolites. The chemical characterization of wastewater was performed before and after treatment, after 30 days of using stationary zeolite as support. The chemical composition of wastewater was evaluated in terms of the products of nitrification/denitrification processes. The greatest ammonium (NH_4_^+^) adsorption was obtained for wastewater contaminated with different concentrations of ammonium, nitrate and nitrite. The wastewater quality index (WWQI) was determined to assess the effluent quality and the efficiency of the treatment plant used, showing a maximum of 71% quality improvement, thus suggesting that the treated wastewater could be discharged into aquatic environments. After 30 days, NH_4_^+^ demonstrated a high removal efficiency (higher than 98%), while NO_3_^+^ and NO_2_^+^ had a removal efficiency of 70% and 54%, respectively. The removal efficiency for metals was observed as follows (%): Mn > Cd > Cr > Zn > Fe > Ni > Co > Cu > Ba > Pb > Sr. Analysis of the microbial diversity in the zeolite samples indicated that the bacteria are formed due to the existence of nutrients in wastewater which favor their formation. In addition, the zeolite was characterized by SEM and the results indicated that the zeolite acts as an adsorbent for the pollutants and, moreover, as a support material for microbial community formation under optimal conditions. Comparing the two studied zeolites, NZ1 (particle size 1–3 mm) was found to be more suitable for wastewater treatment. Overall, the natural zeolite demonstrated high potential for pollutant removal and biomass support for bacteria community growth in wastewater treatment.

## 1. Introduction

Biological processes that remove nitrogen from wastewater generally involve the transformation of different species of nitrogen into gaseous nitrogen, which is then released into the atmosphere without major risks [1,2]. Biological wastewater treatment plants are based on two processes: nitrification and denitrification. Both processes take place in the presence of bacteria growth and are maintained in the presence and in the absence of oxygen, respectively [3,4]. In the nitrification process, ammonium nitrogen is oxidized by autotrophic bacteria (also called ammonia oxidation bacteria) to nitrite. Nitrous-oxidizing autotrophic bacteria oxidize nitrite to nitrate. Denitrification transforms NO_3_-N into elemental nitrogen. Because both nitrates and nitrites are toxic, they must be removed from wastewater [3,5]. The introduction of a combination of growth media and biological reactants into a wastewater treatment plant aims to improve and increase the capacity of the purification process [5,6].

In the last 10–15 years, there has been interest in the use of growth systems by adding suspended substrates to activated sludge, due to the repeated elimination of activated sludge at the time of settling. Any agent or combination of agents that can improve or extend the operating range for pilot installations, as well as for flow installations, will increase the profitability of the installation [7,8]. Various studies and research have been conducted on the use of zeolites in the biological processes of wastewater treatment and anaerobic digestion. Zeolites are porous aluminosilicates with a crystalline structure and have many uses, including as sorbents for pollutants, in catalytic processes, and as fertilizer additives [9]. Natural zeolites are used as adsorbents for many pollutants such as heavy metals (cooper, lead, nickel, cobalt, and arsenic), chlorinated volatile organic compounds, antibiotics, dye, humic acid, and phenolics [10]. Gani et al. [11] reported the use of natural zeolite for simultaneous sulfur dioxide and mercury removal during combustion of coal.

Most of them are used for removing pollutants after activation of their structure by reaction with acids, base, calcination or modification with inorganic salt (NaCl, CaCl_2_, BaCl_2_, NH_4_Cl, FeCl_3_) or cationic surfactant. Modification of natural zeolites was performed in order to increase their adsorption capacity [12]. Currently, nanomaterials are used for wastewater treatment for the removal of contaminates, such as inorganics, organics, and microorganisms. Different types of nanomaterials are used for wastewater treatment, including various metal nanoparticles for degradation of dye-contained wastewater [13], colloidal solutions of silver nanoparticles used for environmental safety [14], silver nanocrystals stabilized with Ag^+^ and Ag^0^ for treatment of azo-contaminated wastewater [15]. In addition, Vellaichamy et al. [16] used composites of polyaniline/manganese dioxide/titanium dioxide (PANI/MnO_2_/TiO_2_) for Cr^6+^ reduction from wastewater. Advantages of zeolites include low cost, and greater availability in comparison to nanomaterials which are synthesized by physical, chemical and biological methods.

There are over 40 types of natural zeolites and there are over 100 types of synthetic or modified zeolites. In order to improve the performance of natural zeolites, various synthetic and modified zeolites can be produced for more applications. Synthetic zeolites are mainly produced by alkali treatment of silica and alumina with materials of synthetic, natural or waste origin [17]. A variety of zeolites (NaA, NaX, NaY, NaP1, Kchabazite, Linde F, ZSM-5, and ZSM-48) have been synthesized using clay minerals as an aluminosilicate source, while others have been sourced from waste materials, such as coal fly ash, rice husk ash, oil shale ash, municipal solid waste, and incineration ash [18]. Several studies have compared the adsorption performances of natural and synthesized zeolites, and their adsorption characteristics depending on their ion-exchange capacity [19,20].

Zeolites can be used successfully to improve the performance of wastewater treatment plants, and can provide a stabilizing effect, both in the short term and for the sedimentation of activated sludge and bacterial mass [21,22]. They not only act as agents that favor the settling of sludge but also as supports for the growth of bacteria, thus fulfilling a function similar to that of a suspended bacterial growth system. Zeolitic material functions as a weighting agent, a substrate and structural unit for bacterial growth. In wastewater treatment, the cultivation of bacteria assimilated to the composition of wastewater influences the performance of the treatment process. In order to obtain a high level of water purification, it is mandatory to determine the optimal amount of zeolite to be used [22]. This amount should not be too large because the bacteria must grow and create a culture on the zeolitic material. This is directly related to the retention time of the solids. Skleničková et al. [23] used zeolites as filters in fish-breeding recirculation systems and studied their effects on nitrifying bacteria. According to their study, zeolites are able to remove more ammonium cations than the nitrifying bacteria used in biological filters—such as *Nitrosomonas* and *Nitrospira* species. Additionally, Perez et al. [24] used natural zeolite as support for treatment of synthetic swine wastewater in an expanded granular sludge bed (EGSB) reactor for 255 days, which resulted in a positive influence on microbial community formation.

Yang [25] studied the use of new biotechnology for co-immobilization of nitrifying bacteria and zeolite in a batchwise fluidized bed to enhance biological nitrification under different ammonia concentrations. According to their work, the presence of ammonia positively influences the nitrification process when at concentrations lower than 50 mg/L.

The microbial community present in biological wastewater treatment plants contains aerobic, anaerobic and facultatively aerobic bacteria. Lipid analysis can give the composition of the microbial community. The adhesion of bacteria to various surfaces has received a lot of attention. Phospholipid fatty acids (PLFAs) can be used to determine the microbial community and estimate the bacteria species in one single analysis by measuring specific substances [26,27,28]. PLFAs were identified by identifying each fatty acid (FA) peak based on retention time during their separation in the gas chromatograph. However, limited studies exist about the use of zeolite as biomass for inoculated bacteria, and the structure and composition of microbial communities.

Generally, soil analysis tests for biomarkers of the microbial community. Each FA has a specific signature for a functional group within the microbial community.

This study was focused on zeolite material used for biological purification and nitrifying bacterial products. PLFA analysis was used to identify the microbial communities produced during the biological purification of wastewater. The presence of each species of microbial community was evaluated and expressed as fungi, Gram-negative bacteria, Gram-positive bacteria, actinomycetes, arbuscular mycorrhizal fungi and microeukaryotes. The quality of wastewater before and after purification was evaluated.

## 2. Materials and Methods

### 2.1. Zeolite Materials

The zeolite used in this research was a natural clinoptilolite-rich zeolite with two particle sizes of 1–3 mm (NZ1), and <10 µm (NZ2), respectively. The natural zeolite was obtained from Racos, Brasov County, Romania. The raw zeolite was crushed and sieved to obtain the desired sizes, and then washed several times with distilled water to remove impurities. The obtained zeolites were activated by heat treatment at 200 °C for 4 h.

### 2.2. Chemicals

All the used chemicals were of analytical reagent grade. Hydrochloric acid (37%), nitric acid (65%), nitrite (1000 mg/L), nitrate (1000 mg/L) and ammonium solutions (1000 mg/L) were purchased from Merck (Darmstadt, Germany). Fatty acid methyl esters (FAME), purchased from Merck (Darmstadt, Germany), were used to confirm the presence of biomarkers as aerobic, anaerobic and facultatively aerobic species. All solutions were prepared using ultrapure water (18.2 MΩ cm^−1^ at 20 °C) obtained from a Direct-Q3 UV Water Purification System (Millipore, Molsheim, France).

### 2.3. Solution Tested in the Used Equipment for Wastewater Treatment

In order to identify the ability of zeolitic material to purify wastewater, raw wastewater and wastewater contaminated with different concentrations of ammonium, nitrate and nitrite, dye, metals were tested. Volumes of 1 L aqueous solutions were used in each experiment, and 100 g of zeolite were used as adsorbents. The chemical solutions used to perform the experiments are presented in Table 1.

**Figure 1:**
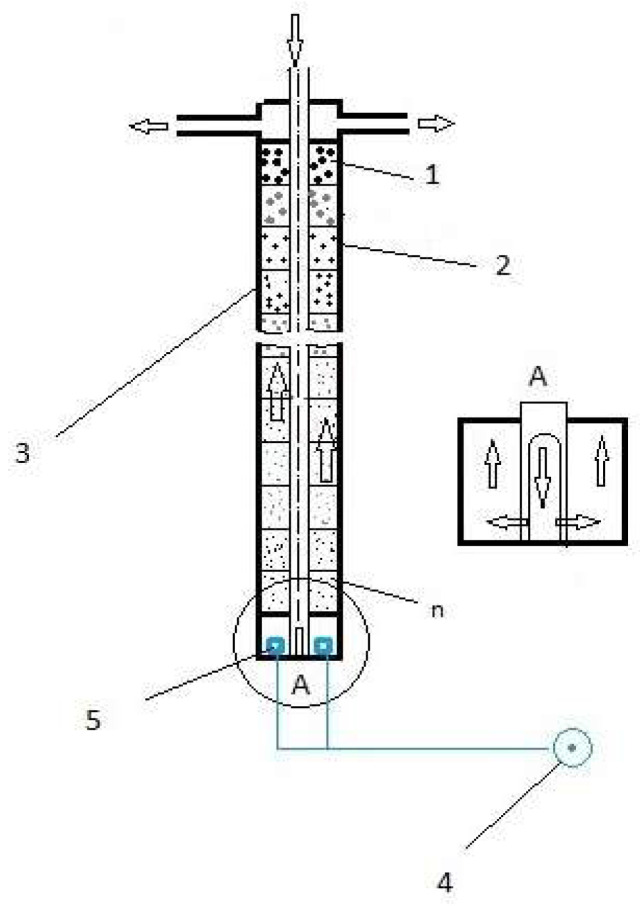
Schematic representation of biological purification of wastewater based on using the zeolite as biomass support.

The equipment used for wastewater purification consists of: 1—zeolite material, 2—submodules (filter components), 3—cylinder filled with zeolite (constitutes the filter of the equipment), 4—compressed air source and 5—compressed air diffusers (according to the schematic representation in Figure 1). The activated zeolites were introduced in each of the cylindrical-shaped elements of the submodules. A tubular cylindrical shape perforated at the bottom was placed crossing the submodules, allowing a downward circuit of water without moistening the zeolite filter material. When wastewater enters the lower submodule of the filter, the flow is ascending through the zeolite mass in each submodule of the cylinder, with aeration favoring the ascending circulation of water through the filter. The compressed air source located at the bottom of the filter ensures the supply of compressed air to the air diffusers. The device was ventilated by air circulation for 30 days. The equipment used for wastewater purification was a joinder between a moving bed biofilm reactor (MBBR) and a sequencing batch reactor (SBR) generally used for treatment of wastewater. The equipment can realize both nitrification/denitrification processes in the same enclosure by altering the aeration of the elements that contain the zeolite.

The proposed mass of zeolite was immersed in real and contaminated wastewater for 30 days, under continuous aeration provided by a compressed air pump. The air flow was maintained at 0.5 L/min during the experiments. All solutions were analyzed to determine the concentrations of ammonium, nitrites and nitrates.

### 2.4. Chemical Characterization of Wastewater

The pH and electrical conductivity (EC) of wastewater were determined using a Seven Excellence multiparameter (Mettler Toledo, Switzerland) after 1 h of settling. Specific metal co (Mn, Al, K, Ca, Na, Fe, Mg, Cu, Pb, Zn, Ni, Cd, and Cr) content was determined by the digestion of samples with a mixture of 65% HNO_3_ and 37% HCl at a volume ratio of 1:3 in a closed polytetrafluoroethylene (PTFE) vessel, using a microwave digestion system (Speedwave MWS-3+, Berghof, Eningen, Germany). The resulting solutions were measured using inductively coupled plasma—optical emission spectrometry (ICP-OES, 5300 Optima DV, Perkin Elmer, Whaltham, MA, USA) [29]. Anionic surfactants from wastewater were determined according to EN 903 [30]. Anion (NO_2_^−^ and NO_3_^−^) content was measured by ion chromatography using a 761 IC compact ion chromatograph (Metrohm, Herisau, Switzerland) according to standard ISO 10301-1 [31]. The wastewater samples were filtered through 0.45 μm polytetrafluoroethylene membrane filters to eliminate the solid particles [31]. Ammonium (NH_4_^+^) content was determined with a Lambda UV–Vis Spectrophotometer (Perkin Elmer, Waltham, MA, USA) applying the salicylate–hypochlorite method [32]. Biological oxygen demand (BOD) was determined according to ISO 5815 [33] and chemical oxygen demand (COD) was determined according to ISO 6060 [34].

### 2.5. Chemical Characterization of Zeolites

In order to determine the cation exchange capacity (CEC), the zeolites were treated with 35 mL sodium acetate 1 N for 5 min (the procedure was repeated for five times), according to the US EPA [35]. Excess sodium acetate was washed out with ethylic alcohol, and Na content was measured by an inductively coupled plasma optical emission spectrometer (ICP-OES). Mn, Al, K, Ca, Na, Fe and Mg content was determined by ICP-OES after the digestion method. Si content was determined by the SEM-EDX method using a scanning electron microscope (SEM VEGAS 3 SBU, Tescan, Brno-Kohoutovice, Czech Republic) with an EDX detector. Loss of ignition (LOI) was determined by the calcination of samples at 550 °C in an oven.

### 2.6. Structural Characterization of Zeolites

#### 2.6.1. Scanning Electron Microscopy (SEM) Analysis and Adsorption Porosimeter Analysis

SEM VEGA 3 SBU with a EDX detector was used to determine the particle size and the morphology of the NZ1 and NZ2 samples. A semiquantitative chemical analysis of zeolites was performed in order to determine the concentration of Si. The specific surface area (S_BET_), volume pore (V_p_), and average pore radius (r) were obtained from N_2_ adsorption–desorption isotherms (measured at −196 °C) using the BET method and the Dollimore–Heal model for porosity data with a Sorptomatic 1990 apparatus (Thermo Electron Corporation, Waltham, MA, USA).

#### 2.6.2. TGA/DTG Analysis

Thermogravimetric analysis (TGA/DTG) of zeolites was conducted with a SDT O 600 (TA Instruments, New Castle, DE, USA), at a temperature range from 30 to 1000 °C, at 10 °C per minute. Under a nitrogen atmosphere, a mass of 5.942 ± 0.3 mg of the dried samples was used for the test. The experiments were repeatable with a standard deviation in peak temperature value.

### 2.7. Determination of Nutrient Removal and Wastewater Quality Index

The amounts of nitrate (NO_3_^−^) and nitrite (NO_2_^−^) in the zeolite samples used as zeolite support were measured before and after purification. Before each analysis, the samples were filtered through a 0.22 µm syringe filter. Removal efficiency (E) was calculated using the Equation (1) [36].

(1) $$E\;(\%)=\frac{C_{I}-C_{F}}{C_{I}}\times 100$$

where *C_I_* is the initial concentration of the parameter (nitrite, nitate, ammonium), and *N_F_* represents the final concentration of the parameter.

The quantity of ammonia removed confirms the nitrification process. The removal efficiency for each parameter found in wastewater was estimated based on the concentration of the parameter at time 0 and at time t = 30 days.

To assess the improvement in wastewater quality, the wastewater quality index (WWQI) was computed. The WWQI is an efficient mechanism to express the overall condition of wastewater that cumulatively describes the quality of multiple chemical parameters [37]. After treatment, wastewater should have a relatively low WWQI, indicating that release into surrounding water bodies is safe. The WWQI is calculated in four steps. In the first step, for each of the 13 physicochemical parameters considered (pH, NH_4_^+^, NO_2_^−^, NO_3_^−^, P, Cu, Pb, Zn, Mn, Ni, Cd, Cr and MBAS), a relative weight (*w_i_*) on a scale of 1 to 5 is assigned based on their critical health effects. The second step is to calculate the relative weight *W_i_* and establish the quality rating *q_i_* (Equations (2) and (3)), followed by calculation of the subindex *SI_i_* for each indicator (Equations (4)), and computing of the WWQI (Equation (5)) [38,39].

(2) $$W_{i}=\frac{w_{i}}{\sum_{i=1}^{n}w_{i}}$$

(3) $$q_{i}=\frac{C_{i}}{S_{i}}\times 100$$

(4) $$SI_{i}=W_{i}\times q_{i}$$

(5) $$WWQI=\overset{n}{\underset{i=1}{\sum }}SI_{i}$$

where *w_i_* denotes each parameter (3 for pH, NH_4_^+^ and MBAS; 4 for all the other metals; 5 for NO_3_^−^ and NO_2_^−^), *W_i_* is the relative weight, *q_i_* is the rating for each parameter, *C_i_* is the measured concentration, *S_i_* is the guideline value according to the drinking water quality guidelines established by Romanian Government Decision no. 188/2002 for the approval of some norms regarding the conditions for discharging wastewater into aquatic environments [40], and *SI_i_* represents the subindex of each parameter [41].

### 2.8. Phospholipid Fatty Acid (PLFA) Analysis

At the end of the experiment (day 30), the zeolite sample was collected from the reactor and used for microbial community identification. The microbial community found on the zeolite surface was determined by the standard PLFA method. The extraction method was realized according to Kovacs et al. [42]. The fatty acids methyl esters were identified based on the MIDI Sherlock^TM^ Microbial Identification System (Microbial ID, Inc., Newark, DE, USA).

### 2.9. Statistics

The statistical processing of the data was performed using OriginPro Data Analysis and Graphing Software (OriginLab Corporation, Northampton, MA, USA), and the Tukey method in order to determine the differences between the varieties. Significance was declared at *p* < 0.05 for all statistical analyses. The a, b, c, d, e, f, g, h, i, j and k letters indicate statistically significant differences at *p* < 0.05.

## 3. Results and Discussion

### 3.1. Chemical Characterization of Zeolites

Zeolites are extremely natural and important materials, with advantages such as low cost of exploitation, easy to prepare, and an exceptional surface area of contact with water compared to unit of volume, making their use as supports for microbial community formation in wastewater treatment a reliable and affordable method.

**Figure 2:**
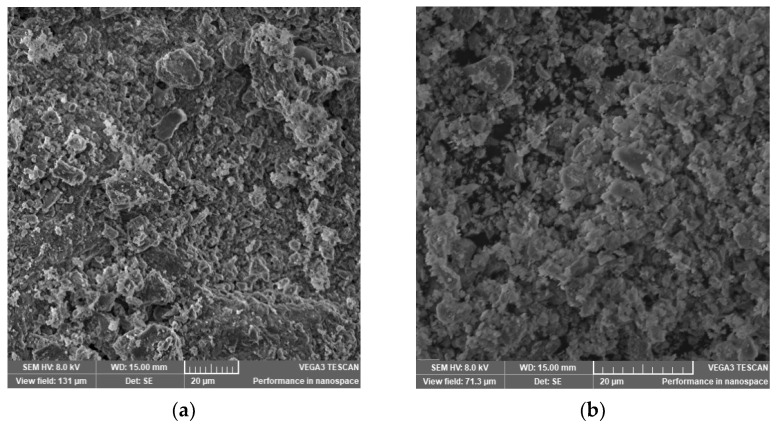
SEM images of (**a**) NZ1 and (**b**) NZ2.

Physicochemical characterization and comparison of the studied zeolites are provided in Table 2, and SEM images are presented in Figure 2. The analyzed zeolites contained the following oxides: SiO_2_, Al_2_O_3_, K_2_O, CaO, Fe_2_O_3_, Na_2_O, and MgO. CEC was much higher for powdered zeolite (1.32 meq/g) than for granulated zeolite. A BET analysis was performed of both the zeolites used in the experiments. S_BET_ and Vp are important parameters for adsorption capacity. The results showed that NZ2 has the largest specific surface area, with a pore volume of 0.261 ± 0.001 cm^3^/g. The average pore radius was 21 Å for NZ2 and 25 Å for NZ1. It was observed that fine granulation (NZ2) resulted in a higher pore volume, which can cause the blockage of zeolite pores. According to Chong et al. [43], an increase in the of activation temperature of zeolites to 400–600 °C led to a reduction in BET surface area. Thus, a temperature of 200 °C was chosen as optimal for zeolite activation and wastewater purification. The chemical composition showed that SiO_2_ and Al_2_O_3_ are the predominant oxides. Small quantities of K_2_O, CaO, Fe_2_O_3_, Na_2_O and MgO were found [44].

### 3.2. Structural Characterization of Zeolites

A SEM analysis was performed to determine the morphology of zeolites. The SEM images revealed two different structures: the structure of NZ2 is porous, whereas the structure of NZ1 is more compacted. The obtained activated structures of zeolites are recommended for the purification of wastewater.

**Figure 3:**
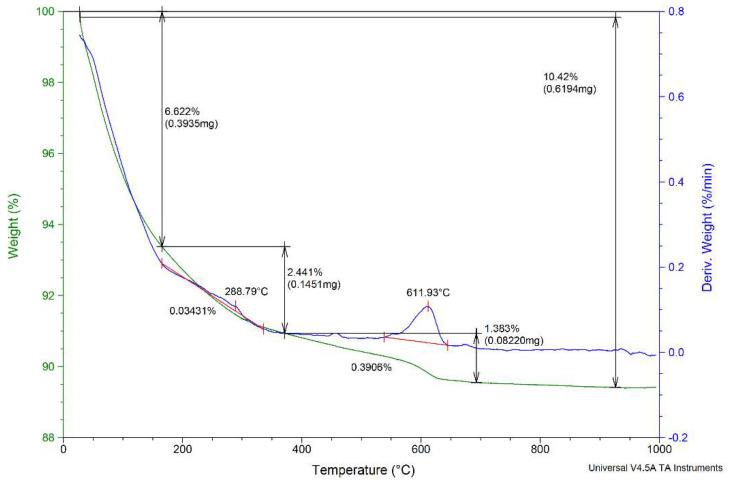
The TGA/DTG analysis of the NZ1 zeolite sample.

The TGA/DTG curves of the NZ1 zeolite are presented in Figure 3. The zeolite was heated from room temperature to 1000 °C at a rate of 10 °C min^−1^ in an N_2_ atmosphere. TGA analysis showed three regions depending on the activation temperature values of the zeolite samples: the first region, from room temperature to 200 °C, is attributed to evaporation of water; the second region, between 200 and 400 °C, corresponds to dealumination of zeolites. According to Garcia-Basabe et al. [45], dealumination of natural zeolite begins before 150 °C and was associated with the lack of extra-framework cations and the strongly bound water molecule within the pore. Another explanation could be the depolymerization of T–O–T bonds (T = Si, Al) and the appearance of the soluble silicates [46]. The third region is the domain 400–800 °C where significant loss of biomass occurs. This temperature range could be associated with the decomposition of zeolite at high temperatures. The activation of natural zeolites was realized using a rigid framework including pores and channels created by a tetrahedral TO_4_ structure. The thermal activation of zeolites at 200 °C enhanced pore volume by removing the water molecules.

### 3.3. Operational Performance of Wastewater Purification and Water Quality Index

Due to the varied chemical composition, biological purification of wastewater in the presence of zeolites is relatively difficult. In this regard, research has been conducted to evaluate the efficiency of biological purification of wastewater using zeolites as a biomass support for microbial community formation.

Table 3 shows the results obtained from the analysis of the wastewater sample before and after zeolite support use (30 days).

Parameters that indicate the degree of contamination of wastewater are pH, solid suspensions, BOD and COD. The removal efficiency of COD in S2 was 66.8% and 79.1% in the case of BOD, indicating organic matter consumption. 

The presence of non-biodegradable substances is highlighted by analysis of nitrogen (in the form of ammonia, nitrates and nitrite), salts (sulfites, sulfates and chloride), metals and hard biodegradable substances (cyanides and benzene, toluene, ethylbenzene and xylenes (BTEX)) [47]. The change in pH from 7.4 ± 0.1 to 8.2 ± 0.64 could be attributed to the oxidation of organic substances present in wastewater. During incubation, the pH of the medium increased and could become more electronegative, due to the formation of bacteria biofilm of on the surface of the zeolites and the possibility of their further growth according to Cayetano et al. [48], who identify biofilm formation as enhancing anaerobic digestion.

Among the negative charges of zeolites and bacteria in the process of their adhesion to zeolites, there are certain repulsions due to their hydrophobicity. The conductivity of 1284 ± 85.7 µS/cm was based on the existing load carried that moves freely in wastewater. In the nitrification process, zeolite can be used as an ion exchange material due to its ability to remove ammonium ions from wastewater, along with its ability to carry biofilm. According to the bioregeneration mechanism of zeolites, the microorganisms are attached to the surface of the zeolites and are trapped in the zeolite powder particles with which they form a microbial biofilm. This microbial zeolite biofilm adsorbs ammonia. Denitrification can occur if electron donors are present. In the aerobic phase, the adsorbed ammonium is released into the liquid phase due to the chemical equilibrium and transformed from nitrite to nitrate, after which nitrate is transformed into gaseous nitrogen and brought back into the atmosphere [49,50].

**Figure 4:**
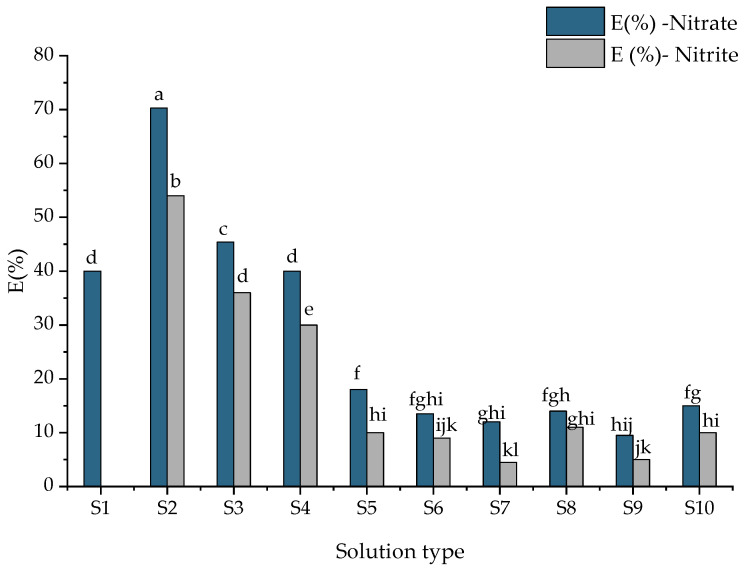
Nitrate and nitrite removal efficiency (E, %) depending on sample types. Data are the mean ± standard deviation (*n* = 3). Means with different letters (a–k) above the bars indicate significant differences based on Tukey’s test (*p* < 0.05).

**Figure 5:**
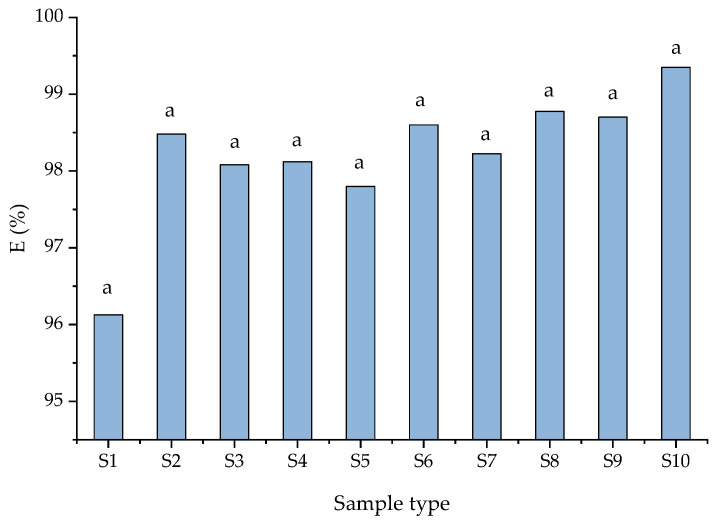
The removal efficiency (%) of ammonium ions depends on sample type. Data are the mean ± standard deviation (*n* = 3) and the same letters show no significant difference (*p* > 0.05).

According to Yang et al. [51], nitrogen removal mechanisms depend on wastewater composition, environmental and operating conditions, such as nitrification and denitrification processes and the possible coexistence of partial nitrification to nitrite or nitrate. Nitrate content in the unpurified wastewater (S1) was 0.5 mg/L while the nitrite was absent. The low removal efficiency of nitrate removal for the S5–S9 samples was attributed to a lack of a source of organic matter. Having in view these results, the concentrations of ammonium (NH_4_^+^), nitrate (NO_3_^−^) and nitrite (NO_2_^−^) were analyzed for each solution tested (Figure 4 and Figure 5). In all cases, the removal efficiency of NH_4_^+^ was higher than 98%. These results indicated that both zeolites were capable of removing NH_4_^+^ from wastewater enriched with different pollutants. An ammonium removal efficiency of 96% was obtained from unpurified wastewater (S1). In the case of using NZ2 zeolite, the removal efficiency has a slightly higher value, maybe due to the granular surface. The nitrate removal efficiency in the S2 samples was 55.0%, whereas the nitrite removal was 46.5%. According to Liu et al. 2022 [1], the high removal efficiency of NH_4_^+^-N promotes biomass growth. The low efficiency of removing nitrates and nitrites from wastewater was attributed to the presence of toxic compounds in sample matrices. The same efficiency of removal was obtained in S3 and S4 (presence of dye). The oxidizing process of ammonia in the presence of dyes was greater than in their absence. This could be due to the presence of favorable conditions (source of organic carbon) for the growth of nitrifying bacteria. The increase in nitrification and denitrification efficiency, respectively, was attributed to the dye that acted as an electron donor if the solution contained nitrites and nitrates. Thus, denitrification is favored by the appearance of heterotrophic bacteria. According to the results presented in Table 3, the concentrations of BOD and COD decreased significantly. The organic substances are considerably reduced due to the adsorption on the surface of the zeolites and the microbial respiration that takes place. The zeolites showed significant differences in nitrification efficiency based on nitrate and nitrite efficiency (presented in Figure 4). According to Kim et al. [52], the zeolite did not need an adaptation period for nitrification and the process can be observed without considering the concentration of ammonia from wastewater. Additionally, the zeolite is regenerated by nitrifiers, and the nitrite and nitrate are desorbed from the zeolite.

Therefore, the results obtained in the present work demonstrate that zeolite particle size does influence ammonium adsorption. High ammonium adsorption was assigned to the effect of air-stripping and nitrification processes carried out in the continuous mode. The greatest removal of ammonia was likely due to zeolite exchanging metal ions with lower charge density cations (such as NH_4_^+^). The sorption mechanism of ammonia was due to cation exchange with Na^+^. The zeolite served dual roles as an ion exchanger and a biomass carrier for bacteria. Comparing the two studied zeolites, NZ1 (particle size 1–3 mm) was found to be more suitable for wastewater treatment. The different ratios of Si/Al from the zeolite framework influence the negative charge of the extended framework. The lower Si/Al ratio increases the negative charge of the zeolite (due to the higher amount of Al^3+^ that replaces Si^4+^ in the zeolite structure) and thus increases zeolite capacity to attract cations.

**Figure 6:**
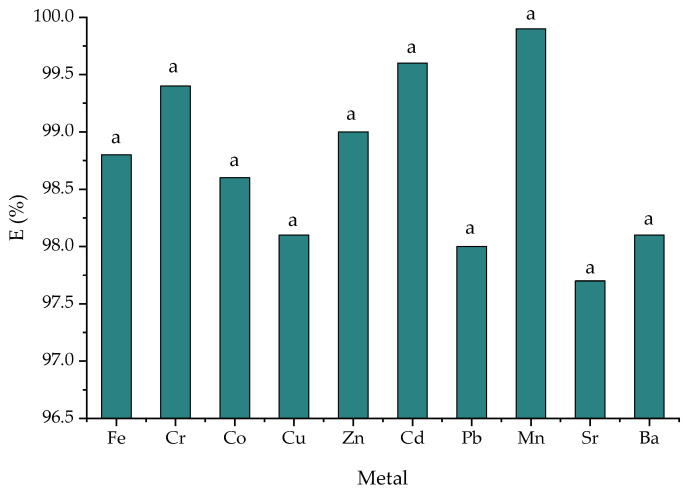
The metal removal efficiency in the S10 sample. Data are the mean ± standard deviation (*n* = 3); the same letters show no significant difference (*p* > 0.05).

Removal efficiency varied as follows (%): Mn > Cd > Cr > Zn > Fe > Ni > Co > Cu > Ba > Pb > Sr (Figure 6). The highest efficiency of 99.6% was obtained for Mn and Cd. The removal of metal ions was due to their adsorption to the structure of zeolites by ion exchange with the interchangeable ions of Na, Ca, K and Mg in the zeolite structure. In the S10 sample, the initial concentration of wastewater was still enriched with another 1 mg/L metal mixture. The final concentrations were Na—66.17 mg/L, Ca—27.9 mg/L. K—7.18 mg/L and Mg—0.782 mg/L. Enrichment with metals such as Na, Ca, K and Mg can be attributed to their presence in the zeolite structure and its ion exchange capacity. According to Gong et al. [53], zeolites can produce strong ion exchange and adsorption effects on heavy metals in solution through the silicon–oxygen tetrahedron and aluminum–oxygen octahedron structures, thus reducing the effectiveness of heavy metals.

Wastewater quality before and after zeolite support purification was expressed as the WWQI and the results obtained are presented in Table 4. In the case of untreated wastewater, the WWQI ranged from 306 to 1050, with a mean value of 756 according to the drinking water standards (DWS) [54]), and between 55.8 and 329, with a mean value of 242, according to the standards for discharging wastewater into aquatic environments (DWAES) Government Decision 188/20.03.2002 for the approval of some norms regarding unloading conditions for discharging of wastewater into aquatic environments [40]. The values obtained for wastewater indices indicate a high level of polluted water before purification. The highest indices were obtained for the S4, S7 and S9 samples, due to the high concentration of ammonium, nitrite and nitrate. After treatment, the WWQI ranged between 27.1 and 401, and 10.4 and 200, indicating that purified wastewater could be released into the municipal network.

After treatment, it was found, by analytical determination, that wastewater was purified with an efficiency of 71%: S1 was of excellent quality; S5, S10, were of good quality, suitable for household activities and irrigation purposes; and S2 was of medium quality, suitable for irrigation purposes without any further treatment.

### 3.4. PLFAs as Biomarkers for the Microbial Community

The PLFA profile of zeolites used as support for bacteria growth was analyzed by gas chromatography with flame ionizing detection (GC–FID). Table 5 summarizes the results of the microbial community analysis. Aerobic bacteria were associated with saturated and hydroxy groups, whereas anaerobic bacteria were associated with unsaturated and branched acids. The facultatively aerobic bacteria also contain unsaturated, branched, and hydroxy fatty acids. The FAME standard was used for the identification of each fatty acid biomarker. According to Quezada et al. [55], fatty acids that indicate eukaryote bacteria are sulfate-reducing bacteria and polyunsaturated fatty acids. Additionally, Gram-positive bacteria contain branched-chain fatty acids, and Gram-negative contain monounsaturated fatty acids. Moreover, actinomycetes contain methyl-branched and unsaturated fatty acids. The analysis of all the microorganisms found on the surface of zeolites used as biomass support for the purification process provided a quantitative description of the microbial community in the aqueous environment. Variation in pollutant concentrations in each solution tested led to the generation of different amounts of microorganisms.

The extraction of fatty acids from the samples was performed with organic solvents (methanol and hexane). After 30 days of incubation, most of the bacteria were immobilized on zeolite. The majority of the bacteria were methanotrophic, anaerobic, arbuscular mycorrhizal and Gram positive. The microbial indices present in each PLFA marker were detailed in Tian et al. [56]. The fatty acid 16:1ω5c was quantified as arbuscular mycorrhizae and quantified as 56.1 ± 3.2 nmol/g zeolite (S2) and 38.6 ± 3.4 nmol/g zeolite (S10). Fungal biomass was quantified as linoleic acid (18:2ω6) content. Other studies reported the use of oleic acid (18:2ω9) as a fungal marker [57,58], but in this study, it was not identified. According to [49], fungi and actinomycetes are responsible for the decomposition of organic residues. 

Bacterial biomass was quantified as the sum of 15:0, 17:0, 15:0iso, 15:0anteiso, 16:0iso, 17:0iso, 17:0anteiso, 16:1ω7, 17:0cyclo, 18:1ω7 and 19:0cycloω8 fatty acids and was quantified as 689.9 ± 71.3 nmol/g zeolite (S2) and 801.5 nmol/g zeolite (S10). The aerobic FAME biomarkers that characterize the Actinomyces bacteria were found in the amount of 53.3 ± 1.4 nmol/g zeolite, while the anaerobic bacteria were found in the amount of 174.5 ± 12.3 nmol/g zeolite. The fatty acids 16:0 10-methyl, 17:0 10-methyl and 18:0 10-methyl were used to quantify Actinomycetes bacteria. The presence of metals in wastewater solution led to a decrease in the concentration of methanotroph bacteria and Arbuscular mycorrhizal, responsible for the denitrification process. The presence of a microbial community was influenced by the composition of wastewater. 

### 3.5. Structural Characterization of Zeolites Used as Biomass Support for Microbial Community Production

**Figure 7:**
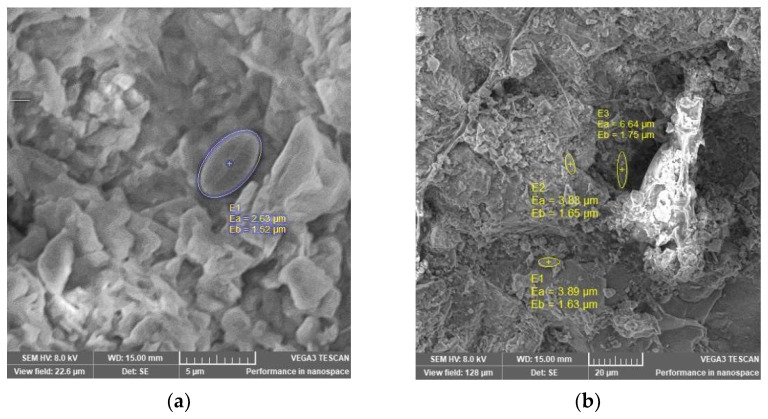
SEM images of NZ 1: (**a**) biofilm of sample S2 at 5 µm and (**b**) biofilm of sample S2 at 20 µm.

SEM micrographs were used to study the biofilm morphologies of zeolite used as support for biofilm. The SEM images of the zeolite used as support for microbial community formation for the S2 samples are shown in Figure 7. The zeolite showed a homogenous morphology with a crystal structure of the granular type of NZ1.

**Figure 8:**
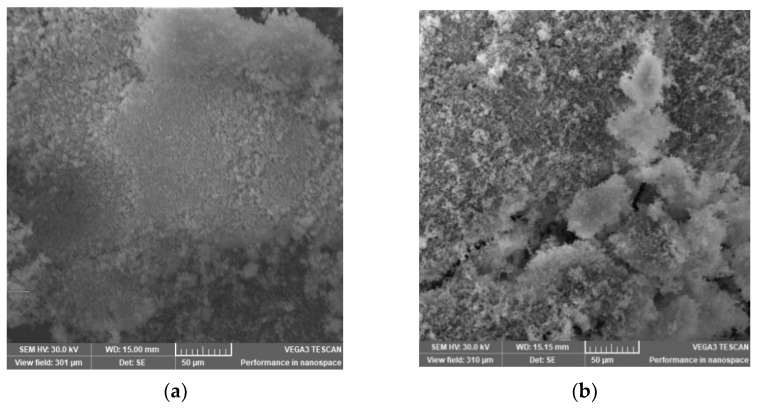
SEM images of: (**a**) biofilm of sample S9 and (**b**) biofilm of sample S10.

The SEM image of the zeolite sample after being used as support for biomass revealed a particle size of less than 5 µm and is characteristic of a cube shape. As shown in SEM images of zeolite, the structure of zeolite used as microbial community support had more pores, protuberances, and a tough-like surface. This structure suggested the capability of zeolite, not only to adsorb the pollutant on an active site, but also to be a better medium to create optimal conditions for the reproduction of microbial bacteria. SEM images of NZ2 after 30 days stationary in the wastewater installation showed an irregular structure with biofilm formation (Figure 8). The photograph of zeolite NZ2 also shows the formation of zeolite floc. 

**Figure 9:**
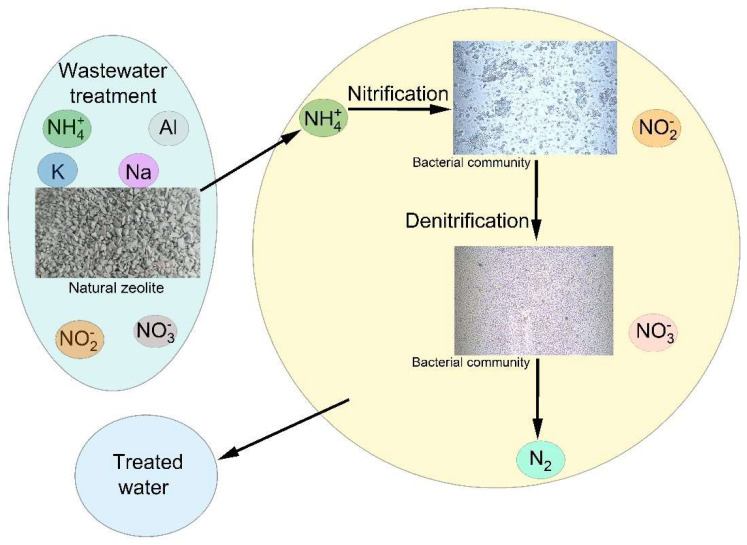
Schematic representation of the mechanism of wastewater treatment.

According to Montalvo et al. [5], addition of a natural zeolite in the biological process enhanced membrane permeability due to the formation of rigid floc with increased nitrification efficiency due to improving the level of microorganism survival. The results showed that the use of zeolites as support for wastewater treatment has many advantages: they can be used as ion exchange materials due to their ability to remove ammonium ions along with their ability to carry biofilm; the permeability of the filter can be considerably increased due to the formation of a rigid structure that has a lower specific resistant than that of the used sludge. The adsorption of pollutants from wastewater and biological purification was the result of multiple mechanisms. The denitrification process was proved by nitrate removal and facultative and anaerobic bacteria are known to be responsible for this process [59]. Nitrate was negatively charged and electrostatic adsorption can explain the observed nitrate removal efficiency. The biological purification of wastewater by a zeolite filter was based on three mechanisms: ion exchange with ammonium, nitrification, and adsorption (Figure 9).

The removal of COD proved that biodegradation of organic contaminates was correlated with nitrifying bacteria.

## 4. Conclusions

This study indicated the potential of using zeolites as biomass support for microbial community formation, and thus purification of wastewater. Wastewater enriched with different concentrations of ammonia, nitrite and nitrate was used to demonstrate the improvement in the nitrification process and the formation of bacteria. Zeolites are carries of bacteria and favor accessibility of microorganisms through cavities. Activated zeolites offer more attachment points for microorganisms. Based on the performed experiments, the use of zeolites in wastewater treatment plants could be a solution for replacing the used sludge or using sludge and zeolite simultaneously. The quality of wastewater determined with the WWQI indicated that the treated wastewater could be discharged into aquatic environments. Microbial communities, identified on zeolite surfaces used for water purification, play an important role in the biological treatment of wastewater, with zeolites being used in ammonia removal from wastewater specifically by ion exchange and capacity for biofilm formation. In conclusion, the implementation of methods in wastewater treatment plants should be tested in order to improve the bioregeneration of zeolites and to reduce the high concentration of contaminants.

## Figures and Tables

**Table 1:** Solutions used for experiments.

Solution Types	Contain	Zeolite	Code
1.	Raw wastewater	NZ1	S1
2.	S1 contaminated with: 50 mg/L ammonia, 50 mg/L nitrite and 50 mg/L nitrate	NZ1	S2
3.	S1 contaminated with: 10 mg/L methylene blue, 50 mg/L ammonia, 50 mg/L nitrite and 50 mg/L nitrate	NZ1	S3
4.	S1 contaminated with: 10 mg/L rhodamine, 50 mg/L ammonia, 50 mg/L nitrite and 50 mg/L nitrate	NZ1	S4
5.	S1 contaminated with: 10 mg/L ammonia, 10 mg/L nitrite and 10 mg/L nitrate	NZ1	S5
6.	S1 contaminated with: 20 mg/L ammonia, 20 mg/L nitrite and 20 mg/L nitrate	NZ1	S6
7.	S1 contaminated with: 40 mg/L ammonia, 40 mg/L nitrite and 40 mg/L nitrate	NZ1	S7
8.	S1 contaminated with: 20 mg/L ammonia, 20 mg/L nitrite and 20 mg/L nitrate	NZ2	S8
9.	S1 contaminated with: 40 mg/L ammonia, 40 mg/L nitrite and 40 mg/L nitrate	NZ2	S9
10.	S1 contaminated with: 1 mg/L metals, 40 mg/L ammonia, 40 mg/L nitrite and 40 mg/L nitrate	NZ2	S10

**Table 2:** Physical and chemical composition (% *w/w*) of zeolites used in the experiments (mean ± standard deviation, *n* = 3).

Parameter	NZ1	NZ2
pH (pH unit)	8.2 ± 0.1 ^e^	8.4 ± 0.1 ^d^
CEC (meq/g)	1.20 ± 0.1 ^h^	1.32 ± 0.1 ^f^
S_BET_ (m^2^/g)	52.85 ± 0.6 ^b^	73.1 ± 2.7 ^a^
V_p_ (cm^3^/g)	0.127 ± 0.001 ^h^	0.261 ± 0.001 ^f^
r (Å)	25.0 ± 0.57 ^c^	21.0 ± 0.50 ^b^
SiO_2_	65.2 ± 2.7 ^a^	72.6 ± 3.1 ^a^
Al_2_O_3_	13.7 ± 1.1 ^d^	14.9 ± 1.1 ^c^
CaO	2.5 ± 0.1 ^gh^	2.54 ± 0.03 ^ef^
K_2_O	2.42 ± 0.1 ^gh^	2.19 ± 0.09 ^ef^
Na_2_O	0.70 ± 0.05 ^h^	0.69 ± 0.01 ^f^
Fe_2_O_3_	1.18 ± 0.6 ^h^	1.02 ± 0.1 ^f^
MgO	0.75 ± 0.05 ^h^	0.65 ± 0.01 ^f^
LOI	6.2 ± 0.41 ^ef^	5.4 ± 0.3 ^de^
Si/Al	4.6 ± 0.15 ^fg^	5.24 ± 0.4 ^de^

LOI—loss of ignition. Different letters of each column showed a significant difference at the level of *p* ≤ 0.05.

**Table 3:** The chemical composition of wastewater before and after purification on zeolite support. (Data represent the mean ± standard deviation; *n* = 3 parallel measurements.)

Parameters	Wastewater (S2)	Wastewater (After Purification)
pH la 20.0 °C	7.4 ± 0.1 ^fg^	8.2 ± 0.64 ^c^
Electrical conductivity (µS/cm)	1300 ± 61.2 ^a^	1284 ± 85.7 ^a^
Suspended solids (mg/L)	19.0 ± 1.3 ^efg^	32.0 ± 0.95 ^c^
Biologycal oxigen demand (BOD)	66.5 ± 2.5 ^cd^	13.9 ± 0.61 ^c^
Chemical oxygen demand (COD)	307 ± 5.5 ^b^	169.0 ± 3.07 ^b^
Ammonia * (NH_4_^+^-N)(mg/L)	64.2 ± 3.2 ^cde^	0.974 ± 0.06 ^c^
Chlorides (mg/L)	89.0 ± 4.1 ^c^	24.0 ± 0.69 ^c^
Nitrite * (NO_3_^—^ mg/L)	50.0 ± 3.4 ^cde^	23.0 ± 0.47 ^c^
Nitrate * (NO_2_^—^ mg/L)	50.5 ± 2.6 ^cdef^	15.0 ± 0.2 ^c^
Phosphates (mg/L)	<0.05	<0.05
Sulfates (mg/L)	23.0 ± 2.2 ^defg^	34 ± 0.98 ^c^
Surfactants (mg/L)	13.5 ± 0.61 ^efg^	1.0 ± 0.06 ^c^
Fosfor total (mg/L)	3.05 ± 0.06 ^g^	0.5 ± 0.01 ^c^
Cooper (mg/L)	0.55 ± 0.06 ^g^	<0.02
Lead (mg/L)	0.52 ± 0.06 ^g^	<0.05
Zinc (mg/L)	1.00 ± 0.07 ^g^	<0.01
Manganese (mg/L)	0.0675 ± 0.001 ^g^	<0.01
Nickel (mg/L)	0.0995 ± 0.0006 ^g^	<0.05
Cadmium (mg/L)	0.0261 ± 0.002 ^g^	<0.02
Cromium (mg/L)	0.0628 ± 0.001 ^g^	<0.05

Note: * The raw wastewater (S1) was contaminated with 50 mg/L of ammonia, nitrite and nitrate. Values indicated with different letters are significantly different from each other at *p*  ≤  0.05 levels, whereas those indicated with the same letters show no significant differences (*p* > 0.05). Columns with different letters show a significant difference at the level of *p* ≤ 0.05.

**Table 4:** Water quality index before and after zeolite purification.

	WWQI			
	Before Purification		After Purification	
	DSW *	DWAES **	DSW *	DWAES **
S1	306	55.8	27.1	10.4
S2	976	329	184	90.2
S3	976	329	311	154
S4	976	329	339	168
S5	440	110	109	52.3
S6	574	165	204	100
S7	842	275	392	195
S8	574	165	202	100
S9	842	275	401	200
S10	1050	383	111	53.7

Note: * DSW represents the guideline values according to the World Health Organization (WHO) [54]; ** DWAES represents the standards for discharging wastewater into aquatic environments [40].

**Table 5:** The microbial community (data represent the mean ± standard deviation; *n* = 3; parallel measurements).

Types	Quantity (nmol/g Zeolite) S2	Quantity (nmol/g Zeolite) S10	FAME Marker
General FAME (bacterial biomass)	689.9 ± 71.3 ^a^	801.5 ± 1.5 ^a^	15:0, 17:0, 15:0iso, 15:0anteiso, 16:0iso, 17:0iso, 17:0anteiso, 16:1ω7, 17:0cyclo, 18:1ω7 and 19:0cycloω8
Arbuscular mycorrhizal fungi	145.2± 14.9 ^b^	12.5 ± 1.31 ^e^	16:1ω5
Methanotrop (Gram negative)	673.5 ± 26.1 ^a^	78.9 ± 6.7 ^de^	18:1ω7c; 17:0cy; 19:0cy
Microeukaryote	98.3 ± 5.5 ^bc^	544.3 ± 31.0 ^b^	20:4ω6c
Fungal biomass	56.1 ± 3.2 ^c^	38.6 ± 3.4 ^e^	18:2ω6
Gram-positive bacteria	106.1 ± 8.8 ^bc^	145.6 ± 9.5 ^cd^	15:0i; 15:0a; 16:0i; 17:0i; 17:0a
Anaerobe bacteria	174.5 ± 12.3 ^b^	192.4 ± 1.0 ^c^	17:0cyclo, 19:0cyclo
Actinomycetes (actinobacteria)	53.3 ± 1.4 ^c^	37.7 ± 3.5 ^e^	16:0 10-Me; 17:0 10-Me; 18:0 10-Me

Note: Values indicated with different letters were significantly different from each other at *p*  ≤  0.05 levels, whereas those indicated with the same letters showed no significant differences (*p* > 0.05). Columns with different letters showed a significant difference at the level of *p* ≤ 0.05.

## Data Availability

Not applicable.
